# Developing and validating of an English questionnaire to assess knowledge, attitudes, and practices regarding Marburg virus disease (EKAP-MVD): A cross-sectional study

**DOI:** 10.1097/MD.0000000000041571

**Published:** 2025-02-21

**Authors:** Mohamed Fakhry Hussein, Shymaa Mamdouh Mohamed Abdu, Assem Gebreal, Fatma Elnagar, Basma E. El Demerdash, Mohamed R. Abonazel, Ayed A. Shati, Saleh M. Al-Qahtani, Ramy Mohamed Ghazy

**Affiliations:** a Occupational Health and Industrial Medicine Department, High Institute of Public Health, Alexandria University, Alexandria, Egypt; b Department of Public Health and Community Medicine, Mansoura University, Faculty of Medicine, Mansoura, Egypt; c Faculty of Medicine, Alexandria University, Alexandria, Egypt; d Health Administration and Behavioral Science Department, High Institute of Public Health, Alexandria University, Alexandria, Egypt; e Department of Operations Research and Management, Faculty of Graduate Studies for Statistical Research, Cairo University, Cairo, Egypt; f Department of Applied Statistics and Econometrics, Faculty of Graduate Studies for Statistical Research, Cairo University, Cairo, Egypt; g Department of Child Health, College of Medicine King Khalid University, Abha, Saudi Arabia; h Family and Community Medicine, College of Medicine, King Khalid University, Abha, Saudi Arabia; i Tropical Health Department, High Institute of Public Health, Alexandria University, Alexandria, Egypt.

**Keywords:** epidemics, general population, knowledge, attitude, practice, Marburg virus disease, Questionnaire validation, Sub-Saharan

## Abstract

Knowledge, attitudes, and practices (KAP) of the general population toward Marburg Virus Disease (MVD) have a crucial impact on control and prevention strategies, particularly during outbreaks. The current study aimed to develop, culturally adapt, and validate a questionnaire for assessing KAP toward MVD (EKAP-MVD). A cross-sectional study using face-to-face interview and an anonymous online survey was conducted from March 13 to April 28, 2023 in 8 Sub-Saharan African countries (Ethiopia, Ghana, Kenya, Lesotho, Nigeria, Senegal, South Africa, and Tanzania). Internal consistency was assessed using Cronbach’s alpha, split-half reliability, and Spearman-Brown coefficient. We assessed EKAP-MVD face and content validity. Construct validity was determined through convergent and discriminant validity, as well as exploratory and confirmatory factor analyses. A total of 510 participants were included: 51.6% were females, 46.5% were aged 18 to 25 years, 65.5% were residents in urban areas, 52.9% did not have university education, 58.6% were single, 34.7% were students, and 15.7% worked in the medical field. The Cronbach’s alpha of the questionnaire was 0.877. All questions showed a statistically significant correlation with their latent factors (*P* < .05), indicating that the questionnaire had good convergent validity. The correlations between domains were either weak positive or negative, indicating discriminate validity. The KMO measure of sampling adequacy for factor analysis was 0.932 and Bartlett’s test of sphericity was significant (*P* < .0001). The elbow point of the scree plot reveals that the number of factors that were most important and should be kept for further analysis was 3. Confirmatory factor analysis model fit was as follows: normed Chi-square (χ^2^) = 1.301, the root mean square error of a pproximation (RMSEA) = 0.038, goodness-of-fit index and comparative fit index > 0.9, and root mean square residual (RMR) < 0.08. In conclusion, the developed questionnaire had good psychometric properties and can be used to assess KAP about MVD.

## 1. Introduction

Marburg virus caused Marburg virus disease (MVD) is a member of the Filoviridae (filovirus) family.^[1]^ It is one of the viral hemorrhagic fevers (VHFs) that cause significant morbidity and mortality and poses a significant threat to human and animal populations in endemic areas.^[2,3]^

Rousettus aegyptiacus fruit bats are thought to be the natural hosts for the Marburg virus, from which it is transmitted to humans. It spreads through direct contact (through broken skin or mucous membranes) with infected people’s blood, secretions, organs, or other bodily fluids, as well as surfaces and materials (e.g., bedding, clothing) contaminated with these fluids. Transmission through contaminated semen can occur up to 7 weeks after clinical recovery. Healthcare workers (HCWs) could be infected while treating patients infected with MVD. Burial ceremonies that involve direct contact with the deceased’s body can also contribute to the spread of this deadly infection.^[1,3]^ The incubation period of MVD ranges from 2 to 21 days. Symptoms start suddenly, with a high fever, severe headache, and malaise. On the third day of symptoms, severe watery diarrhea, abdominal pain and cramping, nausea, and vomiting may occur. Severe hemorrhagic manifestations usually appear 5 to 7 days after the onset of symptoms. Death occurs most frequently between 8 and 9 days after symptom onset and is usually preceded by severe blood loss and shock.^[1,4]^ The case-fatality rate for MVD ranges from 23% to 90%.^[3]^

Following concurrent outbreaks in Marburg, Frankfurt, and Belgrade, MVD was first identified in 1967.^[5]^ The following countries in Sub-Saharan Africa have experienced sporadic outbreaks of MVD: Ghana in 2022; Uganda in 2017, 2014, and 2012; Angola between 2004 and 2005; the Democratic Republic of the Congo between 1998 and 2000; Kenya between 1980 and 1990; and South Africa in 1975. Recently, there have been 9 fatalities and 16 suspected cases of MVD in Equatorial Guinea for the first time as of February 13, 2023.^[1,3]^

These outbreaks lead to human fatalities, morbidities, and strain on the sociocultural and healthcare systems as their control requires a lot of resources, including money, laboratory testing, and personnel. HCWs remained dedicated despite limited resources and a lack of protective equipment in the affected areas. They are often the first to interact with infectious disease patients, are at risk of infection due to a failure to recognize prospective cases, poor infection control, and inadequate healthcare infrastructure.^[6,7]^ Recent research found that 1% to 10% of HCWs who are exposed to MVD became affected.^[8]^ However, a recent Indian study found that only 50.9% of HCWs were knowledgeable about MVD.^[9]^ Therefore, good knowledge about MVD is crucial since it reduces infection risk and promotes cooperation between different sectors. Also, it lets national and international healthcare personnel feel confident despite personal worries.^[10]^

The knowledge, attitudes, and practices (KAP) of the general population toward MVD have a crucial impact on control and prevention strategies, particularly during outbreaks. Human transmission can be decreased by increasing public awareness about Marburg infection. Unfortunately, during previous outbreaks, harmful information was distributed on social media and among individuals, impeding efforts to stop the infection’s spread.^[11–13]^

Every disease epidemic is a chance to raise awareness and educate people how to protect themselves. For a better response to these VHFs outbreaks, previous studies used tools to assess KAP^[14]^ in various African countries based mainly on Ebola virus disease (EVD). (10–12) Moreover, a recent study by Ghazy et al^[15]^ validated a French tool to assess the KAP toward MVD in Sub-Saharan Africa. However, it was noted that Sub-Saharan African nations lacked a specific, validated English tool for evaluating the KAP of MVD. To use a standardized tool for KAP assessment in Sub-Saharan African countries, the current study aimed to develop, culturally adapt, and validate an English questionnaire to assess KAP toward MVD (EKAP-MVD). This essential step in validating a KAP tool concerning MVD within the general population will enhance the nation’s preparedness for potential outbreaks, identify areas for effective training program development and preparedness strategies, improve infection control measures, and ultimately safeguard HCWs and the broader community.

## 2. Materials and methods

### 2.1. Study design and setting

A cross-sectional study was conducted from March 13 to April 28, 2023. The study was conducted in randomly selected 8 English speaking Sub-Saharan African countries (Ethiopia, Ghana, Kenya, Lesotho, Nigeria, Senegal, South Africa, and Tanzania).

### 2.2. Sample size and sampling method

Based on the sample size guidelines of 10 participants per item for questionnaire validation (ratio 10:1),^[14]^ we needed 310 participants. A priori computation of sample size requires 200 samples for confirmatory factor analysis (CFA) using structural equation modeling (SEM).^[16]^ Our analysis required 510 English-speaking participants. Convenience snowball sampling was employed to obtain the necessary sample size from the selected sub-Saharan African countries. Each country had one representative data collector. Data collectors received standardized online training. Collaborators from the selected countries were recruited through the Global Researcher Club, a nonprofit organization dedicated to conducting health-related research across various countries.

### 2.3. Inclusion criteria

Adults who were at least 18 years old, could read English, and were residents in one of the 8 selected countries of the Sub-Saharan African region during the study were eligible to join the study.

### 2.4. Data collection tool

The data was gathered through face-to-face interviews (200 participants) and through an anonymous online survey that was disseminated via email, Facebook, Twitter, and WhatsApp after its submission to a Google form (310 participants). The questionnaire was developed based on previous literature^[1,3,5,16,17]^ and consisted of 4 sections. The first section was for socio-demographic data including (age, gender, nationality, residence, marital status, occupation, and level of education) and information about MVD awareness (hearing about MVD before), source of information, previous marburg virus infection, mode of transmission, working habits changed due to fear of MVD, and frequency of physical contact with others. The second section assessed the knowledge of the respondents about MVD. Knowledge questions covered topics such as the causes, transmission, risk factors, symptoms, prevention, and control of MVD through 15 items. The third section involved 11 items to assess the attitudes of the participants toward MVD. The fourth section consisted of 5 items to assess the practices of the respondents linked to MVD. The questions in sections 2, 3, and 4 were closed-ended with multiple-choice options.

### 2.5. Steps of questionnaire development and validation

#### 2.5.1. Item development

**Figure 1.**
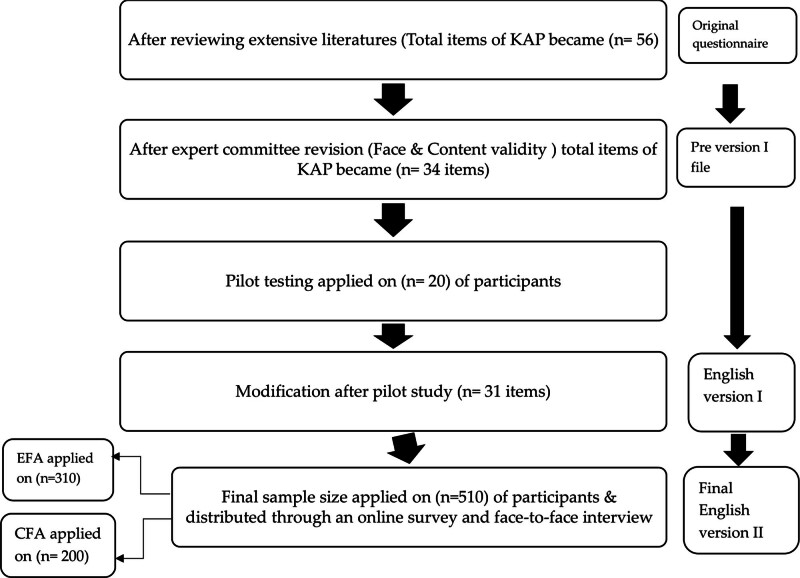
Steps of development and validation of KAP English questionnaire toward Marburg virus disease.

1. Following the extensive literature review, we compiled the following: 56 questions consisting of 34 knowledge items, 13 attitude items, and 9 practice items. The collected questions were organized and revised to remove any potential ambiguity. We avoided double-barreled questions, long questions, and questions with negative words. Additionally, we thoroughly revised the questionnaire to warrant a logical flow of items (Origional questionnaire).
2. An expert committee filtered the questions and removed 20 knowledge, 4 attitude, and 3 practice questions. They then rephrased certain items and added 2 questions to the knowledge domain, 2 to the attitude domain, and 1 to the practice domain, resulting in 34 items: 16 knowledge items, 11 attitude items, and 7 practice items.
3. Due to scale measurement and other technical issues, one knowledge question and 2 practice questions were moved to be asked before the KAP questionnaire itself, so the KAP questionnaire distributed to participants to test the tool’s validity included 15 knowledge items, 11 attitude items, and 5 practice items (total 31 items) (Version I). (Table S1, Supplemental Digital Content, http://links.lww.com/MD/O397) (Fig. 1).

#### 2.5.2. Item interpretation

The following values were assigned to the knowledge scores: 0 for no, 1 for don’t know, and 2 for yes. On a Likert scale with a maximum of 5 points, the attitude scores were calculated as follows: 1 = strongly disagree, 2 = disagree, 3 = neutral, 4 = agree, and 5 = strongly agree. The practice scores ranged from 5 (always), 4 (often), 3 (sometimes), 2 (rarely), and 1 (never). The median score was used as an arbitrary cutoff point following the addition of the scores for each KAP domain.

#### 2.5.3. Face validity and expert evaluation

An expert panel of 8 researchers (methodologists, healthcare professionals, public health professionals, and language professionals) evaluated the content’s validity to verify whether the questionnaire accurately measured what it was intended to measure as the next step in the validation process. The expert panel looked at how the items were written, whether they addressed the defined constructs, and whether minor changes were required.

#### 2.5.4. Pilot testing

Next, we examined the prefinal questionnaire. Twenty responses from Uganda, Zambia, and Somalia were obtained after asking the collaborators to distribute the prefinal survey. We tested items in the questionnaire for understanding, readability, language, terminology, and cultural appropriateness, as well as the clarity of the instructions for each component. A few changes were needed to improve the language clarity.

#### 2.5.5. Psychometric analysis

*Reliability and item analysis*: Cronbach’s alpha measured the tool’s internal consistency. Cronbach’s alpha is considered to be satisfactory if it is between 0.70 and 0.80, and if it is above 0.80 it is excellent.^[18]^ We conduct a split-half reliability analysis through the Güttman Split-Half coefficient to evaluate the reliability of the questionnaire by splitting it into two halves and comparing the scores achieved from each half. The Spearman-Brown coefficient estimates the reliability of a measure when the length of the questionnaire is changed.

*Construct validity*: It indicates the “degree to which an instrument evaluates a construct of concern.”^[19]^ Construct validity indicators were assessed by convergent, discriminant (divergent), and structural validity. By measuring the inter-item correlations and the correlation between each item and the mean score of its subscale, convergent validity is produced. When a subscale’s items have a high value of convergence, they are assessing the same underlying construct. By analyzing the factor correlation matrix of the KAP domains, divergent validity was evaluated. Each domain is distinct and measures a different construct if there is little correlation between the elements.^[20]^

*Factorial analysis validity*: We examined the data gathered from 510 participants. Exploratory factor analyses and CFA were used to perform the factor analysis. We randomly divided the participants into 2 groups: 310 for the exploratory factor analysis (EFA) and 200 for the CFA.^[21,22]^

Exploratory factor analysis: The EFA was carried out to determine the structure of the primary factors and to detect the latent factors.^[22]^ To determine how adequate this measure is, the Kaiser-Meyer-Olkin (KMO) sampling adequacy test and Bartlett’s sphericity test were conducted. Factorial analysis can be used if the *P* value of Bartlett’s test is less than 0.05 (the observed variable correlations in the dataset are significantly different from zero), and the KMO statistics have values between 0 and 1, which indicate factor analysis adequacy (KMO ≥ 0.6 low adequacy, KMO ≥ 0.7 medium adequacy, KMO ≥ 0.8 high adequacy, and KMO 0.9 very high adequacy).^[23]^ Additionally, we extracted the number of latent factors using Eigenvalues (>1). The scree plot was created to determine the optimal number of factors to be retained in the EKAP-MVD.^[24]^ The cumulative % of extraction sums of squared loadings of the 3 domains was calculated. The final EFA was done using the principal component analysis with an oblique oblimin rotation to assess the inter-factor correlation matrix of the 31 items and to examine their discriminant validity. A factor loading cutoff value of 0.30 was chosen to decide which items were highly associated with a given factor. If the *P* value is less than .05, it means that the variables have enough association to move further with component analysis. Version II (Table S2, Supplemental Digital Content, http://links.lww.com/MD/O397).

*Confirmatory factor analysis:* The CFA assessed how well the EFA-identified factor structure fits the observed data. The convergent and discriminant validity of the constructs and model fit measures were evaluated by the SEM technique. The root mean square error of approximation (RMSEA < 0.08), comparative fit index (CFI > 0.9), root mean square residual (RMR ≤ 0.08), and goodness-of-fit index (GFI > 0.9) were used as model fit indicators.^[25]^ Also, normed Chi-square (χ^2^) was used for testing CFA. It was accepted between researchers being less than 2 to less than 5.^[25–27]^ Convergent validity was accepted if the average variance extracted (AVE) values^[28]^ of the different factors were above 0.5. Discriminant validity was confirmed if the square root of AVE for each construct was higher than the inter-correlation between the factors. Moreover, being a latent factor with a construct reliability (CR) of 0.7 indicates good reliability.^[18,29]^

### 2.6. Data management and statistical programs used

Responses were pooled into an online spreadsheet, and then the data was sent to IBM Statistical Package for Social Sciences (SPSS) version 26 (IBM Corp., Armonk, NY), which was used to code and analyze the data. The mean and standard deviation were used to summarize quantitative variables for normally distributed data.^[24]^ The QQ plot and Kolmogorov-Smirnov test were used to examine the normality of the data. Frequency and percentages were used to represent qualitative variables. IBM SPSS software is used to calculate the metrics of reliability, validity analysis, and EFA. IBM SPSS AMOS (version 26) was used to calculate CFA.

### 2.7. Ethical considerations

The study was approved by the Ethical Committee of the High Institute of Public Health- Alexandria University, Egypt (IRB No.: 00013692, serial number: AU0923214330). The research purpose and its related ethical considerations were stated on the first page of the electronic questionnaire. Participants were informed they were free to accept or refuse to participate in this study because their participation was voluntary. The ethical consent form was asked before starting the questionnaire. They were also informed that they could withdraw from the study at any time without any consequences. They were assured that the obtained data would be used only for research purposes, that their data would be confidential, and that the survey was anonymous. The research followed the guidelines set out in the Declaration of Helsinki for medical research involving human subjects.^[30]^

## 3. Results

### 3.1. Characteristics of the study participants

As shown in Table 1, more than half of the participants were females (51.6%). Most of the respondents were 18 to 25 years of age or 26 to 35 years of age (46.5% and 27.8%, respectively). Sixty percent of respondents were recruited from each of the following countries: Nigeria, Senegal, South Africa, and Kenya. About two-thirds of the study participants were residents in urban areas (65.5%), 52.9% did not have university education, 58.6% were single, 34.7% were students, and 15.7% worked in the medical field. Concerning the source of information about MVD, 39.4% of the respondents heard about it from mass media, 36.3% of them heard about MVD from health workers, and 46.3% did not hear about it before. Surprisingly, 16.3% of the studied sample stated that they had previous MVD. Regarding the source of infection, 22.9% of the participants chose the correct answer (bats), and more than half of them did not know the answer (59.6%). Nearly one-quarter of the sample population (19.2%) modified their habits during work because of their fear of getting MVD in the last 3 months. As for the frequency of bodily contact with others, 22.5% of the respondents had never contacted others, and 77.5% of them had frequent physical contact.

**Table 1:** Baseline characteristics of the study population and awareness about MVD (N = 510)

Baseline characteristics	Frequency
	N (%)
Gender	
Male	247 (48.4)
Female	263 (51.6)
Age in years	
18–25	237 (46.5)
26–35	142 (27.8)
36–50	86 (16.9)
**≥** 51	45 (8.8)
Age in years (Mean ± SD)	**30.8 ± 12.3**
Nationality	
Ethiopia	52 (10.2)
Ghana	46 (9.0)
Kenya	67 (13.1)
Lesotho	58 (11.4)
Nigeria	97 (19.0)
Senegal	69 (13.6)
South Africa	71 (13.9)
Tanzania	50 (9.8)
Residence (country)	
Ethiopia	50 (9.8)
Ghana	46 (9.0)
Kenya	67 (13.2)
Lesotho	57 (11.2)
Nigeria	97 (19.0)
Senegal	69 (13.5)
South Africa	74 (14.5)
Tanzania	50 (9.8)
Nature of residential area	
Desert area	10 (2.0)
Forest area	18 (3.5)
Mountainous area	28 (5.5)
Rural area	120 (23.5)
Urban area	334 (65.5)
Education	
I did not complete any level of education	44 (8.6)
Before university education	270 (52.9)
University education	147 (28.8)
Post graduated	49 (9.7)
Social Status	
Married	177 (34.7)
Single	299 (58.6)
Widow	16 (3.2)
Divorced	18 (3.5)
Occupation	
Medical or paramedical fields like physician, nurse, midwife, or healthcare	80 (15.7)
Engineer, manager	29 (5.7)
Farmer, herdsman, fisherman	32 (6.3)
Service and sales workers, trader	53 (10.3)
Student	177 (34.7)
Not working/retired	32 (6.3)
Others	107 (21.0)
Source of information about MVD*	
Health care worker	185 (36.3)
Mass media	201 (39.4)
Community leaders	110 (21.6)
Friends or neighbors	139 (27.3)
Family member	115 (22.5)
Scientific books or scientific websites	142 (27.8)
I did not hear about it before	236 (46.3)
Had previous marburg infection	
No	427 (83.7)
Yes	83 (16.3)
Marburg is easily transmitted from animal-to-human, through direct contact with	
Bats	117 (22.9)
Cattle	21 (4.1)
Mosquitoes	17 (3.4)
Pigs	10 (2.0)
Poultry	15 (2.9)
Rodents	16 (3.1)
Sheep and goats	10 (2.0)
I do not Know	304 (59.6)
Performed modifications in working habits because of the fear of getting MVD	
No	412 (80.8)
Yes	98 (19.2)
Frequency of physical bodily contact with others (handshake etc.)	
Never	115 (22.5)
Once per day	41 (8.0)
Twice per day	52 (10.3)
Three times per day	46 (9.0)
4–6 times per day	118 (23.1)
7–9 times per day	62 (12.2)
10 times per day or more	76 (14.9)

* Not mutually exclusive question; MVD: marburg virus disease.

### 3.2. Reliability analysis and convergent validity

Table 2 displays some descriptive statistics (mean ± SD) for each item as well as the reliability and convergent validity of the EKAP-MVD. The questions (K1–K8, K10–K14) for knowledge displayed different mean scores, ranging from 1.69 ± 0.83 to 1.99 ± 0.93. For all knowledge items, the item-mean score correlations were positive and significant *P* < .001, indicating a consistent correlation between the individual item scores and the overall knowledge score. The Spearman-Brown coefficient equals 0.915. This shows that the reliability of the measure would stay largely unchanged regardless of whether the questionnaire’s length was extended or shortened. The Güttman Split-Half coefficient equals 0.916, representing a high level of internal consistency between the questionnaire’s two halves.

**Table 2:** Descriptive statistics, reliability, and convergent validity of EKAP-MVD

Variable	Mean ± SD	Item-mean score correlation	Cronbach’s alpha	Split half reliability
Knowledge			0.953	Spearman-Brown Coefficient = 0.915 Guttman Split-Half Coefficient = 0.916
K1	1.79 ± 0.89	0.71 (*P* < .001)		
K2	1.69 ± 0.83	0.65 (*P* < .001)		
K3	1.78 ± 0.91	0.81 (*P* < .001)		
K4	1.69 ± 0.84	0.73 (*P* < .001)		
K5	1.86 ± 0.92	0.79 (*P* < .001)		
K6	1.74 ± 0.87	0.76 (*P* < .001)		
K7	1.71 ± 0.83	0.78 (*P* < .001)		
K8	1.78 ± 0.91	0.77 (*P* < .001)		
K10	1.85 ± 0.91	0.82 (*P* < .001)		
K11	1.88 ± 0.92	0.78 (*P* < .001)		
K12	1.99 ± 0.93	0.73 (*P* < .001)		
K13	1.93 ± 0.93	0.75 (*P* < .001)		
K14	1.79 ± 0.88	0.78 (*P* < .001)		
Attitude			0.781	Spearman-Brown Coefficient = 0.722 Guttman Split-Half Coefficient = 0.626
A1	3.58 ± 1.39	0.54 (*P* < .001)		
A3	3.53 ± 1.31	0.61 (*P* < .001)		
A4	3.7 ± 1.27	0.57 (*P* < .001)		
A8	3.47 ± 1.19	0.49 (*P* < .001)		
A10	3.63 ± 1.28	0.58 (*P* < .001)		
Practice			0.601	Spearman-Brown Coefficient = 0.676 Guttman Split-Half Coefficient = 0.630
P1	3.47 ± 1.12	0.45 (*P* < .001)		
P2	3.42 ± 1.25	0.51 (*P* < .001)		
P3	2.7 ± 1.21	0.38 (*P* < .001)		
Overall Cronbach’s alpha			0.877	

The items (A1, A3, A4, A8, and A10) showed various mean scores for attitude, ranging from 3.47 ± 1.19 to 3.63 ± 1.28. The item-mean score correlations were also positive and significant at 0.001 for all attitude variables. The Spearman-Brown coefficient equals 0.722, and the Güttman Split-Half coefficient equals 0.626. Concerning practices, questions (P1–P3) had mean scores ranging from 2.7 ± 1.21 to 3.47 ± 1.12. The item-mean score correlations were positive and significant at 0.001 for all practice variables. The Spearman-Brown coefficient was 0.676 and the Güttman Split-Half coefficient was 0.630. Item to item correlation is shown in Supplementary Digital Content (Tables S1–S3, Supplemental Digital Content, http://links.lww.com/MD/O397).

The Cronbach’s alpha for each of the domains “Knowledge,” “Attitude,” and “Practice” were 0.953, 0.781, and 0.601, respectively. The overall Cronbach’s alpha of the questionnaire was 0.877. The high Cronbach’s alpha values indicate strong internal consistency reliability for the questionnaire. All questions showed a statistically significant correlation with their factors, and all items within each factor had a significant positive correlation (*P* < .001) (Table 2), indicating that the questionnaire had good convergent validity. Inter-item correlation for each sub-scale was highly significant (*P < *.001) (Tables S3–S5, Supplemental Digital Content, http://links.lww.com/MD/O397).

### 3.3. Divergent validity (discriminant validity)

There was a positive correlation between knowledge and attitude (0.043), a positive correlation between knowledge and practice (0.340), and a positive correlation between attitude and practice (0.086). There were no correlation coefficients larger than 0.7; hence, the factors derived from EFA revealed adequate discriminant validity (Table 3).

**Table 3:** Factor correlation matrix of the KAP regarding MVD

	Knowledge	Attitude	Practice
Knowledge	1	0.043	0.340
Attitude	0.043	1	0.086
Practice	0.340	0.086	1

### 3.4. Exploratory factor analysis

**Figure 2.**
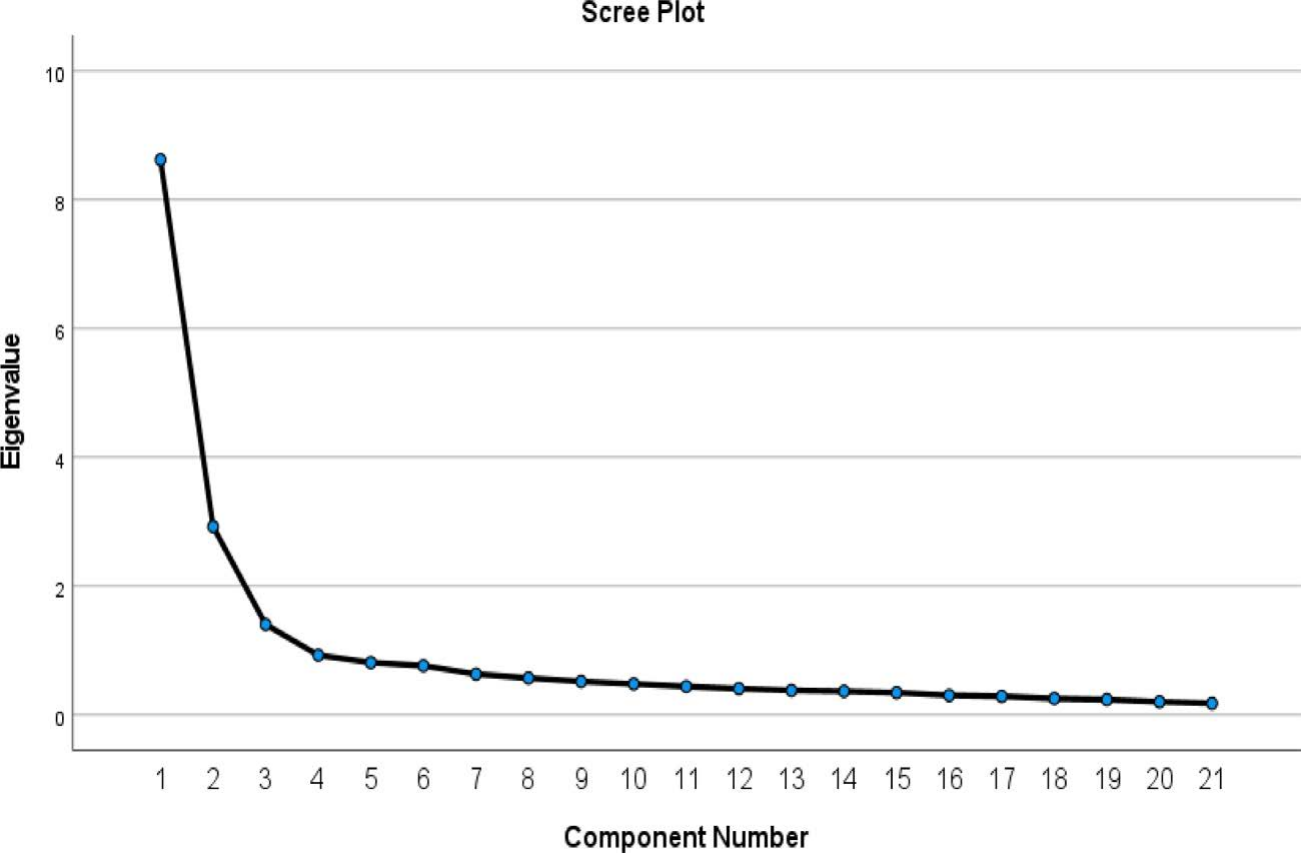
Scree plot of the factors of the EKAP-MVD. The eigenvalues are expressed on the y-axis, while the number of factors is expressed on the x-axis.

The KMO value of 0.932 suggests that the data was suitable for factor analysis due to its high level of adequacy. With 210 degrees of freedom and an approximate χ^2^ value of 3744.354, Bartlett’s test of sphericity produced a *P* value of .0001. This suggests that an identity matrix and a correlation matrix were significantly different from each other, augmenting the use of factor analysis. The first 3 components appear to be the most significant, as indicated by the sharp “elbow” bend in the curve after these components, while the remaining components (4-21) show very small eigenvalues and contribute minimally to explaining the variance in the data. This suggests that retaining 3 components would be sufficient to capture the most important underlying structure of the data while effectively reducing its dimensionality (Figure 2).

Following EFA, 10 items were removed, including 2 knowledge questions, 6 attitude questions, and 2 practice questions. Consequently, the structural matrix revealed that 21 of the items had a high loading on the proposed relevant factors. Version II.

The initial Eigenvalues showed that 21 items of the questionnaire explained 61.64% of the variance in the 3 extracted factors (cumulative % of extraction sums of squared loadings of the 3 factors is 61.64%). For “knowledge,” thirteen items were loaded on one factor, with loading ranging from 0.69 to 0.85. For “attitude,” 5 items were loaded on one factor, with factor loading ranging from 0.63 to 0.76. For “practices,” 3 items were loaded on one factor, with factor loading ranging from 0.52 to 0.72, as shown in Table 4.

**Table 4:** Factor loadings of KAP regarding the MVD questionnaire

Items	Knowledge	Attitude	Practice
K1	**0.75**	−0.12	0.06
K2	**0.69**	−0.14	−0.05
K3	**0.84**	−0.04	−0.11
K4	**0.77**	−0.15	−0.07
K5	**0.82**	0.07	−0.08
K6	**0.81**	−0.06	−0.16
K7	**0.81**	−0.06	−0.12
K8	**0.80**	−0.20	−0.01
K10	**0.85**	−0.05	−0.07
K11	**0.81**	0.09	−0.09
K12	**0.78**	0.16	0.06
K13	**0.79**	0.10	−0.12
K14	**0.82**	−0.03	−0.01
A1	−0.04	**0.68**	−0.20
A3	−0.06	**0.75**	−0.18
A4	0.10	**0.74**	−0.08
A8	0.16	**0.63**	−0.11
A10	0.06	**0.76**	0.01
P1	0.33	0.37	**0.65**
P2	0.34	0.27	**0.72**
P3	0.33	−0.14	**0.52**

KMO = 0.932, Bartlett’s test of Sphericity χ^2^ = 3744.35, df = 210, *P* value = .0001.

Cumulative Eigenvalues = 61.64%.

Extraction method: a principal component analysis. Rotation method: Oblimin with Kaiser normalization.

Bold value indicates that its item refers to which domain.

### 3.5. Confirmatory factor analysis (CFA)

**Figure 3.**
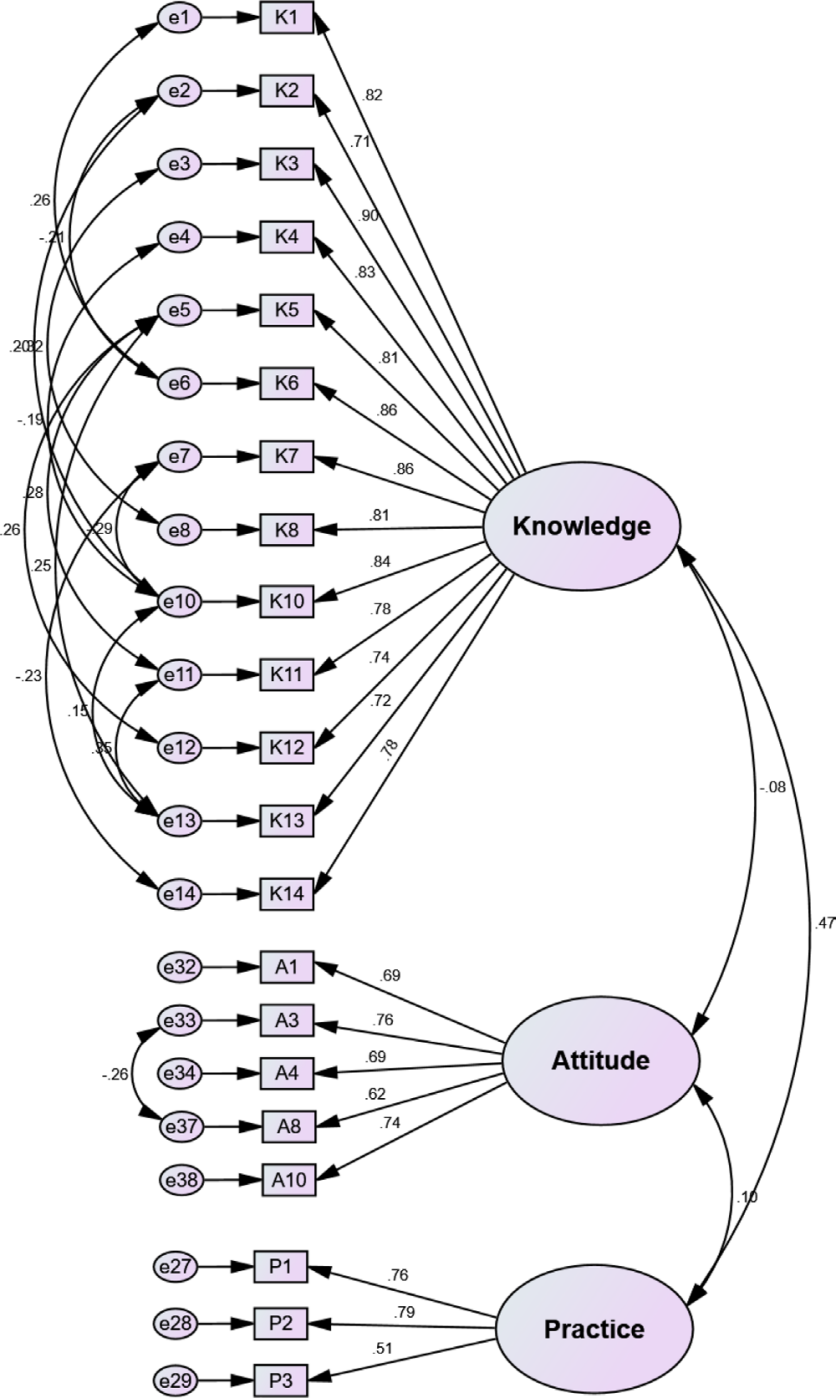
Confirmatory factor analysis of KAP regarding MVD questionnaire.

We ran the CFA on 21 items. The CFA diagram, Figure 3, was constructed to validate the structural relationships between 3 main components (knowledge, attitude, and practice) regarding MVD questionnaire and their respective observable variables. The analysis reveals strong factor loadings across all components, with knowledge items showing particularly robust loadings ranging from 0.71 for K2 to 0.90 for K3, attitude items displaying moderate to strong loadings from 0.62 for A8 to 0.76 for A3, and practice items showing varied loadings from 0.51 for P3 to 0.79 for P2. The correlations between the 3 main factors were also high, suggesting interconnection but distinct constructs, which confirms the theoretical structure of the KAP model and validates the questionnaire’s design for measuring these 3 distinct but related dimensions of MVD understanding.

The main results of the EKAP-MVD confirmatory factor analysis are shown in Table 5. The CR^[31]^ scores represent the latent variables’ internal consistency and reliability (knowledge, attitude, and practice). The CR for the knowledge component was 0.960, the CR for the attitude factor was 0.827, and the CR for the practice factor is 0.736. These numbers imply a high degree of reliability for the latent variables. The degree of variance in the observable variables that were expressed by the latent variables is shown by the average variance explained values.^[28]^ The factor-specific item loadings were acceptable for structural validity since there were no items with loading below 0.3.

**Table 5:** Results of the confirmatory factor analysis of EKAP-MVD. Convergent validity, discriminant validity, and reliability assessment of CFA final model with 3 latent factors and model fit indices

Factor	Construct reliability [16]	Average variance explained [29]	Correlations among latent variables		
			Knowledge	Attitude	Practice
Knowledge	0.96	0.651	0.424*		
Attitude	0.827	0.530	−0.077	0.281*	
Practice	0.736	0.520	0.368	0.099	0.270*
Model fit indices	Normed χ^2^	GFI	CFI	RMSEA	RMR
	1.301	0.910	0.983	0.038	0.070

CFI = comparative fit index, GFI = goodness-of-fit index, RMR = root mean square residual, RMSEA = root mean square error of approximation.

* Diagonal elements are the square root of AVE.

For convergent validity, the AVE^[28]^ values of knowledge, attitude, and practice factors were above 0.5. The AVE for the knowledge factor was 0.651, the AVE for the attitude factor was 0.530, and the AVE for the practice factor was 0.520. These numbers show that a moderate amount of variance in the observed variables can be explained by the latent variables. The correlations between the latent variables are shown by their correlations. knowledge and attitude had a −0.077 correlation coefficient, indicating a weak negative relationship. A moderately positive correlation was found between knowledge and practice, with a value of 0.368. However, the correlation between practice and attitude was relatively weak at 0.099, and it is negative, indicating divergent validity of the tool. The correlation between the 3 latent variables was less than the squared root of AVE, so there is discriminant validity. The model parameters fit were as follow: Normed χ^2^ = 1.301, RMSEA = 0.038, GFI and CFI > 0.9, and RMR* < *0.08.

## 4. Discussion

More than three-quarters of the participants (77.1%) in the current study were either confused about or completely unaware of how MVD is transmitted. According to a previous survey conducted in Uganda, 44.3% of respondents were unaware of how the EVD and MVD are transmitted.^[17]^ This necessitates immediate action to intensify efforts in Sub-Saharan African nations for future studies aimed at assessing KAP regarding MVD. Some Sub-Saharan African cultural traditions promote Marburg virus transmission as a result of cultural practices that may contribute to its spread. All these factors alarm ministries of health in Sub-Saharan Africa to the urgent need for educating populations on transmission, clinical symptoms, reservoirs, control, and prevention.

Researchers can benefit from validation studies since they aid in the evaluation process of studies by pointing out and correcting any sources of bias in the study’s findings. Internal validity refers to the quality of the study itself in terms of its design and the accuracy with which its findings represent the population under study, while external validity refers to the practical relevance of the research’s conclusions, such that the findings can be generalized to similar individuals or populations.^[32]^ Thus, we require several internal-external validation surveys in different nations to identify and close MVD KAP gaps.

This study developed English questionnaire assessing KAP regarding MVD in Sub-Saharan African nationsincluded 3 domains with 21 questions: 13 questions to evaluate knowledge, 5 questions to assess attitude, and 3 questions to assess practices. In the development of the item pool, the authors critically reviewed extensive literature. This ensured the tool content validity at the start of the research.^[1,4,5,17,33]^ The approach would also improve the language and relevancy of the new instrument, which is becoming more applicable for research.^[31]^ Then an expert committee evaluated the items and recommended certain modifications. Moreover, a panel of 8 researchers was selected for their experience in areas of public health, which could support the face and content validity of the questionnaire. We also tested the construct, convergent, and divergent validity of the EKAP-MVD.

Ghazy et al (2024) developed a validated French questionnaire to measure the KAP towards MVD. The questionnaire consisted of 25 questions with a Cronbach’s alpha of 0.87. The other indices for validity indicated the good validity of the tool, which is comparable to our questionnaire.^[15]^

Regarding the psychometric properties of the EKAP-MVD, EFA produced a three-factor structure with 21 items explaining 61.64% of the variance. CFA validated the tool’s EFA-derived factor structures. This study found a satisfactory model-data fit, supporting the factor structure. These results were confirmed by satisfactory RMSEA, CFI, RMR, and GFI^[34]^ and normed χ^2^ of less than 2.^[26]^ This indicates that the model had an acceptable fit.

Considering the reliability of the EKAP-MVD, the tool’s internal consistency determined by Cronbach’s alpha was 0.877. In addition, each domain’s Cronbach’s alpha was greater than 0.7, and the split-half reliability of the questionnaire was goodindicating good internal consistency.^[18]^ There were no correlation coefficients greater than 0.7 between domains (KAP), indicating adequate discriminant validity. Moreover, the CFA confirmed that items within each domain measured the intended domain because they had factor loadings over 0.3. This indicates the existence of structural validity.^[17,33,35]^

Democratic Republic of Congo,^[36]^ Guinea,^[37]^ Uganda,^[17]^ Ghana,^[38]^ Nigeria,^[33]^ and Liberia^[39]^ conducted Ebola Virus Disease (EVD) community studies, although fewer have explored KAP toward MVD.^[17,40]^ A Sierra Leone survey found that participants had a high level of knowledge of EVD but low MVD knowledge.^[40]^ In another Ugandan survey, 51% of them knew how EVD and MVD spread.^[17]^ Each country’s outbreak severity may explain this MVD knowledge gap. This highlights the need for a validated tool to assess KAP regarding MVD as a priority disease^[41]^ to close KAP gaps in Sub-Saharan African communities.

In light of these findings, the EKAP-MVD can be deemed a valid and reliable instrument for assessing KAP in the general population of Sub-Saharan nations concerning MVD. According to the evaluation of EKAP-MVD, public health policies in Sub-Saharan nations can be tailored to improve the awareness of the population toward MVD. Additionally, this tool can measure the KAP of various hemorrhagic virus fevers after some adjustments, opening doors for this underdeveloped area.

### 4.1. Strengths and limitations of the study

This research is the first to create and validate a questionnaire that assesses people’s KAP related to MVD. The results of this study provide an essential basis for further investigation and can guide public health initiatives focused on raising awareness of MVD, modifying attitudes, and encouraging proper behavior. We used different modalities to collect the data (face-to-face interviews and online questionnaires) to ensure the representation of the heterogeneity of the target population. Moreover, we conducted the study in 8 countries to guarantee the questionnaire’s cross-cultural validity. However, this study has some limitations. First, the non-probability sampling technique we used limits how broadly our results can be applied. Caution should be taken when interpreting the results because the sample could not be representative of the total population. Second, we were unable to evaluate the concurrent validity of the tool due to the absence of another valid English instrument for assessing KAP regarding MVD. Finally, we did not calculate the Content Validity Index or conduct test-retest reliability.

## 5. Conclusions

The developed EKAP-MVD had good internal consistency. It also had good convergent and discriminant validity. The structural validity was confirmed using EFA that identified 3 underlying factors and confirmatory factor analysis confirmed the model fit. Thus, EKAP-MVD can be used in the future to assess the KAP of the general population about MVD in Sub-Saharan African nations. This tool can help to identify knowledge gaps, risky behaviors, and inappropriate practices, enabling policymakers to develop targeted interventions that control this disease and prevent future pandemics. Future research should evaluate the external validity of this tool and implement it in other affected communities worldwide.

## Acknowledgments

The authors extend their appreciation to the Deanship of Research and Graduate Studies at King Khalid University for funding this work through Small Research Project under grant number RGP 1/188/45.

## Author contributions

**Formal analysis:** Mohamed Fakhry Hussein.

**Investigation:** Shymaa Mamdouh Mohamed Abdu, Mohamed R. Abonazel.

**Methodology:** Assem Gebreal.

**Resources:** Fatma Elnagar, Ramy Mohamed Ghazy.

**Software:** Basma E. El Demerdash.

**Supervision:** Ramy Mohamed Ghazy.

**Validation:** Ramy Mohamed Ghazy.

**Visualization:** Ayed A. Shati.

**Writing – review & editing:** Saleh M. Al-Qahtani, Ramy Mohamed Ghazy.

## Correction

Due to a production error, this article was originally published with Shymaa Mamdouh Mohamed Abdu’s name listed incorrectly as Ghazy Abdu. The name has now been corrected in the online version.

## Supplementary Material


