# Supplementary material for: Developing and validating of an English questionnaire to assess knowledge, attitudes, and practices regarding Marburg virus disease (EKAP-MVD): A cross-sectional study
Source: Medicine (Baltimore). 2025 Feb 21;104(8):e41571. doi: 10.1097/MD.0000000000041571 (PMC11856897; doi:10.1097/MD.0000000000041571)
Supplement: Supplementary file 1 [file medi-104-e41571-s001.docx]

**Knowledge, Attitude, and practices towards Marburg Virus Disease**

You are invited to participate in research entitled "knowledge, attitude, and practices towards Marburg virus disease among General Population". This information is crucial in designing health education programs and mass media materials before and during outbreaks, which generate better control of upcoming epidemics. It will assess causation, signs and symptoms, transmission, risk factors, prevention, and control of the disease.

Your participation is voluntary and anonymous. You are free to withdraw at any time. This study was approved by the Ethics Committee of the Faculty of Medicine with IRB number (00012098). The information provided will be used only for research purposes. Confidentiality will be maintained. The survey should take about 5 to 10 minutes to complete. Thank you for sharing your valuable time with us.

1. **I accept to be involved in the research.**
   1. Yes, Continue
   2. Do not want to be in the research.
2. **Chose the language.**
   1. English
   2. French

**Please answer the following socio-demographic questions**

1. **Gender or sex**
2. Male
3. Female
4. **Age (by years)**
5. **Nationality**
6. **Now I am living in**
7. **Place of residence**
   1. Urban area
   2. Rural area
   3. Mountainous area
   4. Forest area
   5. Desert area
8. **Level of education completed.**
   1. I did not complete any level of education.
   2. Primary education
   3. Secondary education
   4. University education
   5. Post graduated.
9. **Social status**
10. Married
11. Single
12. Widow
13. Divorced
14. **Occupation**
    1. Medical field or paramedical eld like physician, nurse, midwife or healthcare
    2. Engineer
    3. Chemist
    4. Manager
    5. Clerk
    6. Farmer
    7. Miner
    8. Herdsman
    9. Fisherman
    10. Service and sales workers
    11. Elementary occupations
    12. Student
    13. Trader
    14. Not working / retired
    15. Others
15. **Have you heard about Marburg Virus Disease before?**
    1. Yes
    2. No
    3. I do not know
16. **Source of Information about Marburg Virus Disease**
    1. Health worker
    2. Mass media
    3. Community leaders
    4. Friends or neighbors
    5. Family member
    6. Scientific books or scientific websites
    7. I did not hear about it before

1. **Have you had a Marburg infection before?**
   1. Yes
   2. No
2. **Marburg is easily transmitted from animal-to-human, through direct contact with**
   1. Cattle
   2. Sheep and Goats
   3. Mosquitoes
   4. Bats
   5. Poultry
   6. Pigs
   7. Rodents
   8. I do not know
3. **In the past three months, have you modified your working habits for fear of getting Marburg Virus Disease?**
   1. Yes
   2. No
4. **After you knew About Marburg virus disease (MVD), How frequently do you have physical bodily contact with others (handshake etc.)?**
   1. Never
   2. Once per day
   3. Twice per day
   4. Three times per day
   5. Four times per day
   6. Five time per day
   7. Six times per day
   8. Seven times per day
   9. Eight times per day
   10. Nine times per day
   11. Ten times per day or more

**Questions about your knowledge about Marburg Virus Disease:**

1. **Marburg virus disease (MVD) is known as Marburg hemorrhagic fever**
   1. Yes
   2. No
   3. I do not know
2. **Marburg virus disease (MVD) outbreaks have originated in Africa**
3. Yes
4. No
5. I do not know
6. **Marburg is easily transmitted from human-to-human by direct contact with blood and body fluids**
   1. Yes
   2. No
   3. I do not know
7. **Marburg virus can spread through food.**
   1. Yes
   2. No
   3. I do not know
8. **Individuals at risk for contacting MVD: hunters health workers, contact with patients, travelers to epidemic areas**
   1. Yes
   2. No
   3. I do not know
9. **Marburg virus disease (MVD) causes sever bleeding**
   1. Yes
   2. No
   3. I do not know
10. **Sever Diarrhea and vomiting is one of the Marburg virus disease (MVD) symptoms**
    1. Yes
    2. No
    3. I do not know
11. **Marburg virus disease (MVD) ends by death in half of patients**
    1. Yes
    2. No
    3. I do not know
12. **There was a licensed Marburg virus vaccine available at the time of this study**
    1. Yes
    2. No
    3. I do not know
13. **Avoiding mines and caves where fruit bats thrive can prevent the spread of disease.**
    1. Yes
    2. No
    3. I do not know
14. **Avoiding contact with an infected person or his objects can prevent the spread of disease.**
    1. Yes
    2. No
    3. I do not know
15. **Reporting symptoms of Marburg virus disease (MVD) to local health authorities is * important to prevent further disease transmission**
    1. Yes
    2. No
    3. I do not know
16. **Wearing personal protective equipment like gloves could prevent the disease spread**
    1. Yes
    2. No
    3. I do not know
17. **Avoiding funeral of (MVD) infected patient prevent transmission of the disease from the dead person.**
    1. Yes
    2. No
    3. I do not know
18. **There is a treatment for Marburg virus disease (MVD) at the time of this study**
19. Yes
20. No
21. I do not know

**Questions about your attitude toward Marburg Virus Disease**

**To what extend do you agree with these sentences? Answers will be 5 points Likert scale range from strongly disagree or not at all for "1" and strongly agree or yes of course for "5".**

1. I should learn more about Marburg virus disease (MVD)
2. I worry that Marburg virus disease (MVD) can be transmitted to my country or become epidemic
3. People should be tested when they are in contact with someone infected
4. I think Marburg virus disease (MVD) prevention and control measures are important
5. I should take Marburg virus vaccine if it is available
6. Traveling to Marburg virus disease (MVD)-infected countries should be restricted
7. I worry Marburg virus will become a new pandemic
8. I do trust the information about Marburg virus disease (MVD) from scientific experts
9. I worry that Marburg virus is an attempt to reduce the size of global population
10. I should take more hygienic measures like frequent hand washing
11. Rate your fear of getting Marburg Virus Disease (MVD)

**Questions about your behavior against Marburg Virus Disease:**

1. **How often do you wash your hands at least 20 seconds with soap and water or alcohol-based hand sanitizers?**
   1. Always
   2. Often
   3. Sometimes
   4. Rarely
   5. Never
2. **How often do you wash and disinfect surfaces and utensils?**
   1. Always
   2. Often
   3. Sometimes
   4. Rarely
   5. Never
3. **How often do you wear facemask nowadays?**
   1. Always
   2. Often
   3. Sometimes
   4. Rarely
   5. Never
4. **How often do you wear overshoes nowadays?**
   1. Always
   2. Often
   3. Sometimes
   4. Rarely
   5. Never
5. **How often do you wear gloves nowadays?**
   1. Always
   2. Often
   3. Sometimes
   4. Rarely
   5. Never

**Thanks for your time**

**Knowledge, Attitude, and practices towards Marburg Virus Disease**

You are invited to participate in research entitled "knowledge, attitude, and practices towards Marburg virus disease among General Population". This information is crucial in designing health education programs and mass media materials before and during outbreaks, which generate better control of upcoming epidemics. It will assess causation, signs and symptoms, transmission, risk factors, prevention, and control of the disease.

Your participation is voluntary and anonymous. You are free to withdraw at any time. This study was approved by the Ethics Committee of the Faculty of Medicine with IRB number (00012098). The information provided will be used only for research purposes. Confidentiality will be maintained. The survey should take about 5 to 10 minutes to complete. Thank you for sharing your valuable time with us.

1. **I accept to be involved in the research.**
   1. Yes, Continue
   2. Do not want to be in the research.
2. **Chose the language.**
   1. English
   2. French

**Please answer the following socio-demographic questions**

1. **Gender or sex**
2. Male
3. Female
4. **Age (by years)**
5. **Nationality**
6. **Now I am living in**
7. **Place of residence**
   1. Urban area
   2. Rural area
   3. Mountainous area
   4. Forest area
   5. Desert area
8. **Level of education completed.**
   1. I did not complete any level of education.
   2. Primary education
   3. Secondary education
   4. University education
   5. Post graduated.
9. **Social status**
10. Married
11. Single
12. Widow
13. Divorced
14. **Occupation**
    1. Medical field or paramedical eld like physician, nurse, midwife or healthcare
    2. Engineer
    3. Chemist
    4. Manager
    5. Clerk
    6. Farmer
    7. Miner
    8. Herdsman
    9. Fisherman
    10. Service and sales workers
    11. Elementary occupations
    12. Student
    13. Trader
    14. Not working / retired
    15. Others
15. **Have you heard about Marburg Virus Disease before?**
    1. Yes
    2. No
    3. I do not know
16. **Source of Information about Marburg Virus Disease**
    1. Health worker
    2. Mass media
    3. Community leaders
    4. Friends or neighbors
    5. Family member
    6. Scientific books or scientific websites
    7. I did not hear about it before

1. **Have you had a Marburg infection before?**
   1. Yes
   2. No
2. **Marburg is easily transmitted from animal-to-human, through direct contact with**
   1. Cattle
   2. Sheep and Goats
   3. Mosquitoes
   4. Bats
   5. Poultry
   6. Pigs
   7. Rodents
   8. I do not know
3. **In the past three months, have you modified your working habits for fear of getting Marburg Virus Disease?**
   1. Yes
   2. No
4. **After you knew About Marburg virus disease (MVD), How frequently do you have physical bodily contact with others (handshake etc.)?**
   1. Never
   2. Once per day
   3. Twice per day
   4. Three times per day
   5. Four times per day
   6. Five time per day
   7. Six times per day
   8. Seven times per day
   9. Eight times per day
   10. Nine times per day
   11. Ten times per day or more

**Questions about your knowledge about Marburg Virus Disease:**

1. **Marburg virus disease (MVD) is known as Marburg hemorrhagic fever**
   1. Yes
   2. No
   3. I do not know
2. **Marburg virus disease (MVD) outbreaks have originated in Africa**
3. Yes
4. No
5. I do not know
6. **Marburg is easily transmitted from human-to-human by direct contact with blood and body fluids**
   1. Yes
   2. No
   3. I do not know
7. **Marburg virus can spread through food.**
   1. Yes
   2. No
   3. I do not know
8. **Individuals at risk for contacting MVD: hunters health workers, contact with patients, travelers to epidemic areas**
   1. Yes
   2. No
   3. I do not know
9. **Marburg virus disease (MVD) causes sever bleeding**
   1. Yes
   2. No
   3. I do not know
10. **Sever Diarrhea and vomiting is one of the Marburg virus disease (MVD) symptoms**
    1. Yes
    2. No
    3. I do not know
11. **Marburg virus disease (MVD) ends by death in half of patients**
    1. Yes
    2. No
    3. I do not know
12. **Avoiding mines and caves where fruit bats thrive can prevent the spread of disease.**
    1. Yes
    2. No
    3. I do not know
13. **Avoiding contact with an infected person or his objects can prevent the spread of disease.**
    1. Yes
    2. No
    3. I do not know
14. **Reporting symptoms of Marburg virus disease (MVD) to local health authorities is * important to prevent further disease transmission**
    1. Yes
    2. No
    3. I do not know
15. **Wearing personal protective equipment like gloves could prevent the disease spread**
    1. Yes
    2. No
    3. I do not know
16. **Avoiding funeral of (MVD) infected patient prevent transmission of the disease from the dead person.**
    1. Yes
    2. No
    3. I do not know

**Questions about your attitude toward Marburg Virus Disease**

**To what extend do you agree with these sentences? Answers will be 5 points Likert scale range from strongly disagree or not at all for "1" and strongly agree or yes of course for "5".**

1. I should learn more about Marburg virus disease (MVD)
2. People should be tested when they are in contact with someone infected
3. I think Marburg virus disease (MVD) prevention and control measures are important
4. I do trust the information about Marburg virus disease (MVD) from scientific experts
5. I should take more hygienic measures like frequent hand washing

**Questions about your behavior against Marburg Virus Disease:**

1. **How often do you wash your hands at least 20 seconds with soap and water or alcohol-based hand sanitizers?**
   1. Always
   2. Often
   3. Sometimes
   4. Rarely
   5. Never
2. **How often do you wash and disinfect surfaces and utensils?**
   1. Always
   2. Often
   3. Sometimes
   4. Rarely
   5. Never
3. **How often do you wear facemask nowadays?**
   1. Always
   2. Often
   3. Sometimes
   4. Rarely
   5. Never

**Thanks for your time**

**Appendix 3**

**Table 1.S Inter-Item Correlation Matrix of (Knowledge)**

|  | **K1** | **K2** | **K3** | **K4** | **K5** | **K6** | **K7** | **K8** | **K10** | **K11** | **K12** | **K13** | **K14** |
| --- | --- | --- | --- | --- | --- | --- | --- | --- | --- | --- | --- | --- | --- |
| **K1** | 1 | 0.576 | 0.603 | 0.544 | 0.566 | 0.602 | 0.589 | 0.645 | 0.622 | 0.561 | 0.500 | 0.484 | 0.548 |
| **K2** | 0.576 | 1 | 0.543 | 0.491 | 0.564 | 0.573 | 0.542 | 0.618 | 0.502 | 0.474 | 0.438 | 0.456 | 0.505 |
| **K3** | 0.603 | 0.543 | 1 | 0.669 | 0.647 | 0.689 | 0.704 | 0.683 | 0.710 | 0.634 | 0.568 | 0.623 | 0.657 |
| **K4** | 0.544 | 0.491 | 0.669 | 1 | 0.641 | 0.609 | 0.614 | 0.647 | 0.598 | 0.535 | 0.521 | 0.538 | 0.645 |
| **K5** | 0.566 | 0.564 | 0.647 | 0.641 | 1 | 0.630 | 0.646 | 0.598 | 0.682 | 0.663 | 0.652 | 0.639 | 0.631 |
| **K6** | 0.602 | 0.573 | 0.689 | 0.609 | 0.630 | 1 | 0.705 | 0.659 | 0.636 | 0.613 | 0.533 | 0.587 | 0.601 |
| **K7** | 0.589 | 0.542 | 0.704 | 0.614 | 0.646 | 0.705 | 1 | 0.681 | 0.691 | 0.598 | 0.536 | 0.582 | 0.615 |
| **K8** | 0.645 | 0.618 | 0.683 | 0.647 | 0.598 | 0.659 | 0.681 | 1 | 0.686 | 0.530 | 0.520 | 0.504 | 0.611 |
| **K10** | 0.622 | 0.502 | 0.710 | 0.598 | 0.682 | 0.636 | 0.691 | 0.686 | 1 | 0.724 | 0.632 | 0.648 | 0.672 |
| **K11** | 0.561 | 0.474 | 0.634 | 0.535 | 0.663 | 0.613 | 0.598 | 0.530 | 0.724 | 1 | 0.748 | 0.720 | 0.647 |
| **K12** | 0.500 | 0.438 | 0.568 | 0.521 | 0.652 | 0.533 | 0.536 | 0.520 | 0.632 | 0.748 | 1 | 0.689 | 0.630 |
| **K13** | 0.484 | 0.456 | 0.623 | 0.538 | 0.639 | 0.587 | 0.582 | 0.504 | 0.648 | 0.720 | 0.689 | 1 | 0.732 |
| **K14** | 0.548 | 0.505 | 0.657 | 0.645 | 0.631 | 0.601 | 0.615 | 0.611 | 0.672 | 0.647 | 0.630 | 0.732 | 1 |

Note: **. Correlation is significant at the *p<*0.001 (2-tailed).

**Table 2.S Inter-Item Correlation Matrix of (Attitude)**

|  | **A1** | **A3** | **A4** | **A8** | **A10** |
| --- | --- | --- | --- | --- | --- |
| **A1** | 1 | 0.562 | 0.397 | 0.287 | 0.366 |
| **A3** | 0.562 | 1 | 0.431 | 0.349 | 0.438 |
| **A4** | 0.397 | 0.431 | 1 | 0.408 | 0.478 |
| **A8** | 0.287 | 0.349 | 0.408 | 1 | 0.457 |
| **A10** | 0.366 | 0.438 | 0.478 | 0.457 | 1 |

**Table 3.S Inter-Item Correlation Matrix of (Practice)**

|  | **P1** | **P2** | **P3** |
| --- | --- | --- | --- |
| **P1** | 1 | 0.513 | 0.202 |
| **P2** | .513 | 1 | 0.286 |
| **P3** | 0.202 | 0.286 | 1 |
